# The role of ARHGAP9: clinical implication and potential function in acute myeloid leukemia

**DOI:** 10.1186/s12967-021-02733-5

**Published:** 2021-02-12

**Authors:** Caixia Han, Shujiao He, Ruiqi Wang, Xuefeng Gao, Hong Wang, Jingqiao Qiao, Xiangyu Meng, Yonghui Li, Li Yu

**Affiliations:** 1 Department of Hematology-Oncology, International Cancer Center, Shenzhen University General Hospital, Shenzhen University Health Science Center, 1098 Xueyuan Ave, Shenzhen, 518060 China; 2 Medicine School, Nankai University, 94 Weijin Road, Tianjin, 300071 China

**Keywords:** ARHGAP9, AML, t(15;17), Prognosis, Auto/allo-HSCT, Chemotherapy

## Abstract

**Background:**

Rho GTPase activating protein 9 (ARHGAP9) is expressed in various types of cancers and can inactivate Rho GTPases that mainly regulate cytoskeletal dynamics. However, the exact role of ARHGAP9 in acute myeloid leukemia (AML) has yet to be clarified.

**Methods:**

We compared the transcriptional expression, prognosis, differentially expressed genes, functional enrichment, and hub genes in AML patients on the basis of the data published in the following databases: UALCAN, GEPIA, Gene Expression Omnibus, the Human Protein Atlas, Cancer Cell Line Encyclopedia, LinkedOmics, Metascape, and String. Data from the Cancer Genome Atlas database was used to evaluate the correlations between ARHGAP9 expression and various clinicopathological parameters, as well as the significantly different genes associated with ARHGAP9 expression.

**Results:**

We found that ARHGAP9 expression was higher in the tissues and cell lines extracted from patients with AML than corresponding control tissues and other cancer types. ARHGAP9 overexpression was associated with decreased overall survival (OS) in AML. Compared with the ARHGAP9^low^ group, the ARHGAP9^high^ group, which received only chemotherapy, showed significantly worse OS and event-free survival (EFS); however, no significant difference was observed after treatment with autologous or allogeneic hematopoietic stem cell transplantation (auto/allo-HSCT). The ARHGAP9^high^ patients undergoing auto/allo-HSCT also had a significantly better prognosis with respect to OS and EFS than those receiving only chemotherapy. Most overlapping genes of the significantly different genes and co-expression genes exhibited enriched immune functions, suggesting the immune regulation potential of ARHGAP9 in AML. A total of 32 hub genes were identified from the differentially expressed genes, within which the KIF20A had a significant prognostic value for AML.

**Conclusions:**

ARHGAP9 overexpression was associated with poor OS in AML patients and can be used as a prognostic biomarker. AML patients with ARHGAP9 overexpression can benefit from auto/allo-HSCT rather than chemotherapy.

## Background

Acute myeloid leukemia (AML), which is caused by the malignant transformation of myeloid primordial cells, is the second most common type of leukemia diagnosed in adults and children. Despite the significant progress made in risk stratification, supportive care, multiagent chemotherapy intensification, and autologous or allogeneic hematopoietic stem cell transplantation (auto/allo-HSCT), the outcome for AML patients remains discouraging because of recurrence and refractory [1, 2]. Approximately 10% to 40% of younger patients and a larger percentage of patients aged 60 and above have refractory after standard chemotherapy (40%–60%) [3]. Therefore, identifying robust prognostic markers is crucial to provide optimal care for AML patients.

The Rho family of GTPases is a family of small (~ 21 kDa) signaling G proteins, which act as molecular switches and are tightly controlled by guanine nucleotide exchange factors (GEFs) and GTPase activating proteins (GAPs) by generating active GTP-binding and inactive GDP-binding proteins, respectively [4, 5]. Activated Rho GTPases interact with their downstream effectors to regulate the cytoskeleton of the cell membrane or other cellular compartments [6, 7]. Considerable research has supported the importance of Rho GTPases in hematopoiesis and confirmed that Rho GTPases are related to cytoskeleton rearrangement including adhesion, cytokinesis, differentiation, migration, engraftment, aging, and self-renewal in the cellular process [8–14]. The reorganization of the actin and microtubuleb cytoskeleton is essential for platelet adhesion and thrombus formation to avoid excessive bleeding.

A total of 80 Rho family GAPs have thus far been identified, but fewer than half of them in cancer have been clearly investigated. Rho GTPase activating protein 9 (ARHGAP9) containing RhoGAP, SH3, WW, and PH domains, is a member of the Rho GAPs family. Research indicates that ARHGAP9 suppresses the adhesion of KG-1 (a human leukemia cell line) to fibronectin and collagen via the activation of Cdc42 and Rac1 rather than RhoA [15]. ARHGAP9 is also considered as a MAP kinase docking protein, and the WW domain of ARHGAP9 interacts with the CD domains of Erk2 and p38alpha, leading to the inactivation of MAP kinases [16]. ARHGAP9 inhibits the migration and invasion of hepatocellular carcinoma cell by increasing FOXJ2/E-cadherin expression [17]. By contrast, silencing ARHGAP9 reduces the proliferation, migration, and invasion of breast and gastric cancer cells in vitro [18, 19]. These occurrences suggest that ARHGAP9 plays distinct roles in various physiological conditions or various tissues and cells.

No studies have thus far been conducted to access the expression profile and functions of ARHGAP9 in AML, although ARHGAP9 expression in peripheral blood leukocytes has been demonstrated [15]. In the present study, we investigated ARHGAP9 expression in human AML samples and cell lines, as well as explored its associations with clinicopathological factors. We then we evaluated the prognostic significance of ARHGAP9. We also investigated the differentially expressed genes associated with ARHGAP9 expression and discussed their potential functions in AML.

## Materials and methods

### Analysis of The Human Protein Atlas (HPA) database

The HPA (https://www.proteinatlas.org/) is user-friendly online server, which contains the human transcriptomic and proteomic data in cells, tissues, and organs from human normal or pathological tissues via RNA sequencing (RNA-Seq) analysis and immunohistochemistry (IHC) [20]. Thus, ARHGAP9 expression was analyzed in multiple cell lines, such as various leukemia cell lines, lung cell lines, breast cell lines, and brain cell lines in HPA by using the search term “ARHGAP9”.

### The Cancer Cell Line Encyclopedia (CCLE) database analysis

The CCLE project (https://portals.broadinstitute.org/ccle) is an effort to conduct comprehensive genetic characterization of a large panel of human cancer cell lines from individuals of various lineages and ethnicities [21]. It provides public access to analysis and visualization of mRNA expression, mutation data, DNA methylation, DNA copy number, and histone H3 modification for > 1100 cancer cell lines, such as breast cell lines, gastric cancer cell lines, and AML cell lines. RNA-Seq of RNA expression was used to verify ARHGAP9 expression in various cell lines.

### UALCAN database analysis

The UALCAN database (http://ualcan.path.uab.edu/) contains level-3 RNA-seq and clinical data from 31 cancer types selected by the Cancer Genome Atlas (TCGA) [22]. It is an interactive web resource for the in-depth analysis of RNA-Seq expression. The function module of TCGA analysis in the UALCAN database was used in the pan-cancer analysis of ARHGAP9 expression.

### GEPIA database analysis

The GEPIA database (http://gepia.cancer-pku.cn) is a comprehensive resource for the systematic analysis of gene expression [23]. This database includes 9736 tumor and 8587 normal tissue samples from the TCGA and the Genotype-Tissue Expression (GTEx) projects. In the function module, single-gene analysis was used to create plots for ARHGAP9 expression in various cancers and normal tissues. The threshold was selected as the default value.

### The Gene Expression Omnibus (GEO) database analysis

ARHGAP9 expression profiles in various chromosome abnormalities were acquired from the GEO (https://www.ncbi.nlm.nih.gov/geo) database. Data on ARHGAP9 expression of GSE14468 and GSE13159 were downloaded from the profile graph of the GEO2R online program in the GEO according to the gene ID. A total of 200 and 512 AML patients were from GSE14468 and GSE13159, respectively. The data were the raw counts provided by the submitter.

### The Cancer Genome Atlas (TCGA) database analysis

We studied 151 AML patients with RNA-seq and clinical data from the TCGA (https://portal.gdc.cancer.gov) to analyze the significantly different genes, prognosis, and association between ARHGAP9 expression and different clinicopathologic features [24]. The patient IDs used in the present study are listed in the Additional file 1: Table S1. Specifically, 79 patients received only chemotherapy, whereas 67 patients underwent chemotherapy and auto/allo-HSCT. The main clinical and genetic characteristics of the AML patients are presented in Table 1 and Additional file 1: Table S2. RNA-seq and clinical data are available on the TCGA website. These patients were divided into two groups (ARHGAP9^low^ and ARHGAP9^high^) based on the median values of the ARHGAP9 transcript by using the RNAseq data from TCGA. The Limma package in R 3.3.3 was used to screen significantly different genes between the ARHGAP9^high^ and ARHGAP9^low^ groups in AML. Adjusted P < 0.05 and |log1.2 FC|≥ 1 were used as cut-off values for identifying significantly different genes.

**Table 1:** Correlations between ARHGAP9 expression and clinicopathological feathers in AML from TCGA cohort

Patient characteristics	ARHGAP9 expression		
	**Low (n = 76)**	**High (n = 75)**	**p**
Sex, male/female	38/38	45/30	0.217
Median age, years (range)	53.5 (21–77)	60 (21–88)	0.091
Median BM blasts, % (range)	75 (30–100)	69 (30–99)	0.071
Median WBC, × 109/L (range)	13.35 (0.4–137.2)	27.6 (0.6–223.8)	0.194
Median PB blasts, % (range)	40 (0–97)	39 (0–96)	0.322
FAB classifications			
M0	6	9	0.399
M1	12	24	0.019
M2	14	23	0.067
M3	14	0	0
M4	16	5	0.011
M5	10	1	0.005
M6	1	0	1
M7	1	0	1
NA	1	0	1
Cytogenetics			
Normal	24	37	0.026
t(8;21)	3	4	0.719
t(15;17)	14	0	< 0.001
inv.(16)	7	3	0.327
+ 8	5	3	1
11q23	7	1	0.063
-7/del(7)	2	4	0.442
Complex	5	13	0.041
Others	8	8	0.978
No data	1	2	1
Risk level			
Good	24	7	0.001
Intermediate	34	45	0.060
Poor	17	21	0.453
NA	1	2	1

*n* number of patients, *FAB* French–American–British subtypes, *BM-blast*, bone marrow blast, *PB-blast* peripheral blood blast, *WBC* white blood cell

### LinkedOmics database analysis

The co-expression genes in correlated with ARHGAP9 expression were analyzed using the LinkedOmics database (http://www.linkedomics.orglogin.php) [25]. The LinkedOmics database, including mRNA sequencing data from 173 AML patients from the TCGA database, was used to determine ARHGAP9 co-expression in AML. In the LinkFinder module of LinkedOmics, Pearson’s correlation coefficient was calculated to analyze the data. Volcano plots displaying the results were generated.

Overlapping genes between significantly different genes and co-expression genes were determined using the website Draw Venn Diagram (http://bioinformatics.psb.ugent.be/webtools/Venn/). The overlapping genes were used for the subsequent analysis, including ARHGAP9 functional enrichment and protein–protein interaction (PPI) analysis.

### Functional enrichment and PPI analysis

Metascape (http://metascape.org/gp/index.html#/main/step1) is a free gene-list analysis tool for gene functional enrichment analysis [26]. The identified overlapping genes were inputted into the Metascape database for Gene Ontology (GO), Kyoto Encyclopedia of Genes and Genomes (KEGG) pathways, tissues, and disease enrichment analysis.

We employed the String database (https://string-db.org/) to analyze the PPI network [27], which was visualized using the software Cytoscape_v3.6.1 [28]. Hub genes among the PPI were screened using the Cytoscope plugin MCODE with the following parameters: degree cutoff = 2, node score cutoff = 0.2, k-core = 2, and maximum depth = 100.

### Statistical analysis

Data were Statistically analyzed using IBM SPSS 19.0.0. Pearson’s chi-square and Fisher’s exact tests were selected to compare the categorical variables, such as sex, French–American–British (FAB) classification, and cytogenetics classification. The number of samples was considerably less than 5000 in the two groups; thus, the Shapiro–Wilk test was used to explore whether the values in each group were normally distributed for the comparison of continuous variables. Two-sample Student’s t-test was used if the values in each group were normally distributed; otherwise, the Mann–Whitney U test was used. The continuous variables mainly included age, bone marrow (BM), peripheral blood (PB), white blood cell (WBC) in this study. Except GEPIA and LinkedOmics databases were used to evaluate overall survival (OS) of AML patients. The prognostic effect of ARHGAP9 expression on event-free survival (EFS) and OS were analyzed with the Log–rank and Gehan–Breslow–Wilcoxon test in GraphPad Prism 7.0.

## Results

### ARHGAP9 overexpression in AML cell lines

**Fig. 1:**
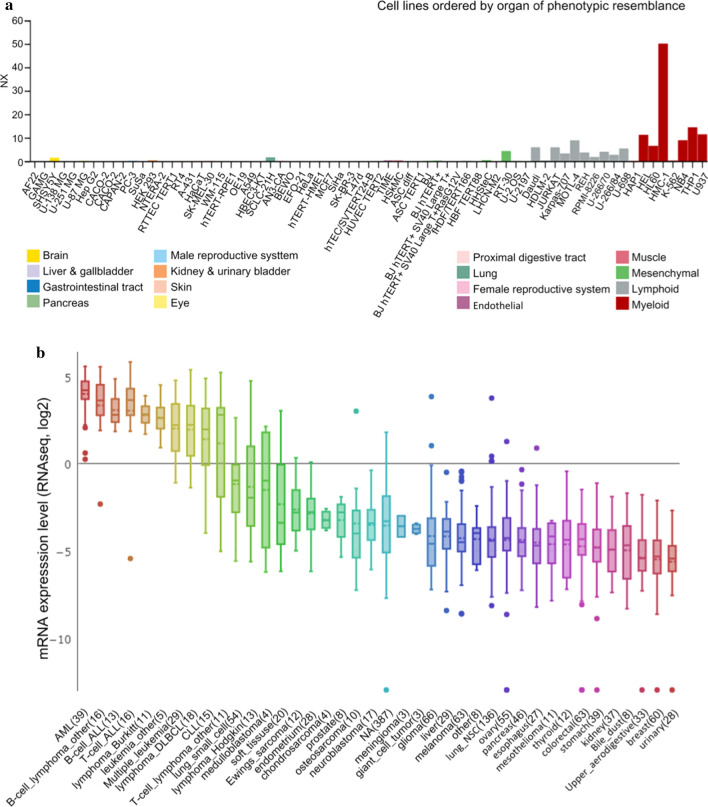
ARHGAP9 expression in cell lines. **a** ARHGAP9 expression in leukemia cell lines, analyzed by HPA. **b** The expression of ARHGAP9 in leukemia Cell Lines, analyzed by CCLE

To elucidate the significance of ARHGAP9 expression in AML cells, we first analyzed ARHGAP9 expression based on the RNA-Seq data obtained from cell lines recorded in the CCLE and HPA databases. In the HPA database, the ARHGAP9 mRNA expression levels were higher in AML cell lines, such as HEL, HL60, NB4, and U937, than that in lymphoid cell lines; meanwhile, ARHGAP9 mRNA was almost not expressed in other cell lines representing the brain, breast, lung and so on (Fig. 1a). Moreover, ARHGAP9 showed the highest expression in AML cell lines in the CELL database (Fig. 1b).

### ARHGAP9 overexpression in AML

**Fig. 2:**
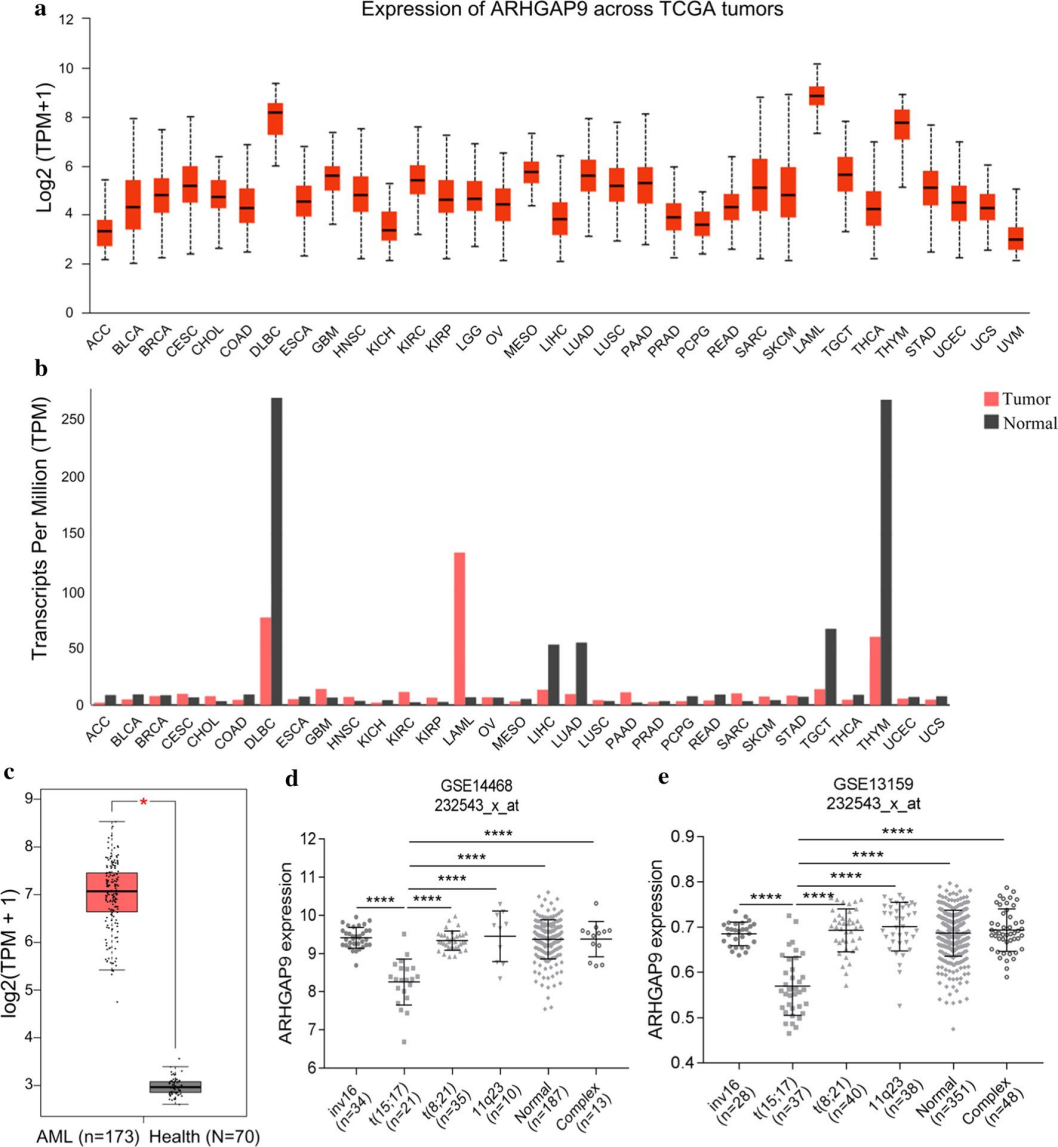
ARHGAP9 expression in AML. **a** ARHGAP9 expression in pan-cancer, analyzed by UALCAN. **b** ARHGAP9 expression in various cancer tissues and normal tissues analyzed by GEPIA; The height of the bar represents the median expression of a certain tumor type or normal tissue. **c** ARHGAP9 expression in AML and normal tissues analyzed by GEPIA. **d** and **e** ARHGAP9 expression in AML with various chromosomal abnormalities in the microarray data of GSE14468 and GSE13159 from the GEO database, respectively

Using the UALCAN and GEPIA databases, we subsequently tested the mRNA expression of ARHGAP9 in different human tumor samples. The mRNA expression levels of ARHGAP9 was the highest among all types of human cancers (Fig. 2a, b). The transcriptional levels of ARHGAP9 in cancers were then compared with those in normal samples by using the GEPIA database. The results indicated that the expression level of ARHGAP9 was significantly upregulated in patients with AML (Fig. 2c). To further study whether ARHGAP9 expression was influenced by different chromosomal abnormalities, we retrieved two microarray data (GSE14468 and GSE13159) from the GEO database and evaluated ARHGAP9 expression among AML patients with major recurrent chromosomal translocations, including inv(16), t(8;21), t(15;17), 11q23, and complex, as well as the normal karyotype. Analysis results of both data sets showed that t(15;17) AML patients exhibited the lowest ARHGAP9 expression among the patients with cytogenetic abnormalities (Fig. 2d, e). These results for the AML samples corresponded with those in the cell lines.

### Relationships between ARHGAP9 and clinicopathological characteristics of patients with AML

In this study, the clinical and molecular characteristics of all patients from the TCGA database are summarized in Table 1. No significant differences in age, sex, BM blasts, WBC, and PB blasts (p > 0.05) were found between ARHGAP9^low^ and ARHGAP9^high^ groups (Table 1). Significant differences were determined in the distribution of FAB classification, cytogenetics, risk stratification, and gene mutations. High expression of ARHGAP9 was significantly correlated with FAB-M1 (p = 0.019), whereas low expression of ARHGAP9 was significantly correlated with FAB-M3 (p < 0.001), FAB-M4 (p = 0.011), and FAB-M5 (p = 0.005) in the distribution of FAB classifications. For cytogenetics, ARHGAP9 overexpression was particularly associated with cytogenetically normal AML (CN-AML) (p = 0.026), and low expression of ARHGAP9 was associated with the t(15;17) (p < 0.01) and complex (p = 0.041) subtypes. Moreover, ARHGAP9^low^ cases tended to be associated with a good prognosis, whereas ARHGAP9^high^ cases were obviously correlated with intermediate risk. Among the mutated genes, high expression of ARHGAP9 was only correlated with WT1 mutation (Additional file 1: Table S2).

### Prognostic values of ARHGAP9 in AML

**Fig. 3:**
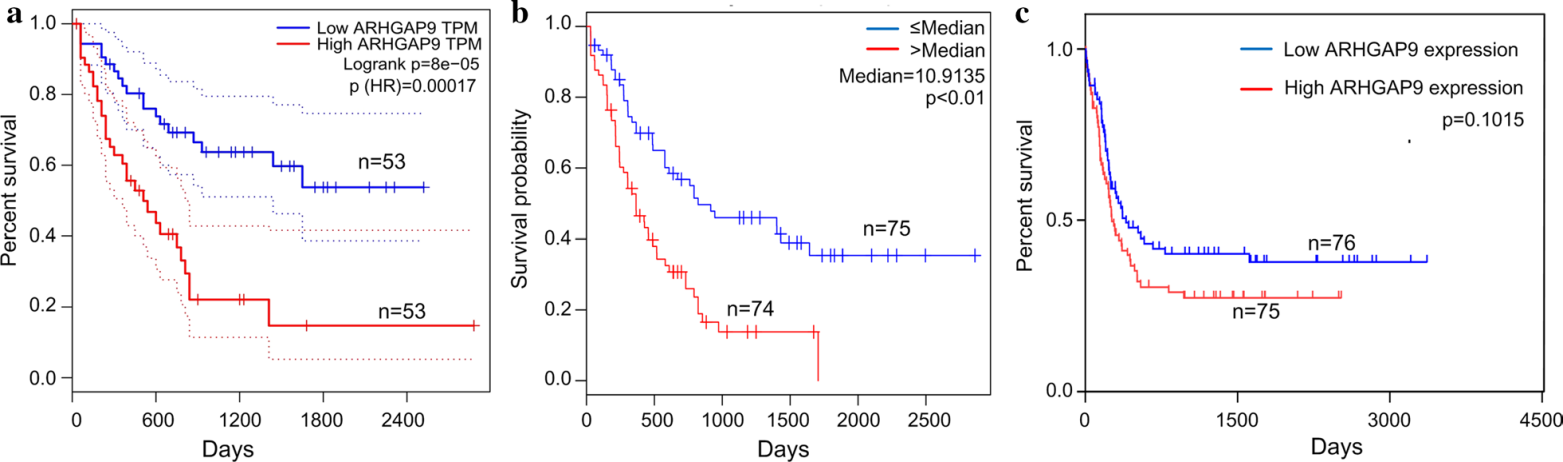
Effect of ARHGAP9 on the survival of AML patients. **a** Prognostic value of ARHGAP9 mRNA level in AML, analyzed by GEPIA. **b** Prognostic value of ARHGAP9 mRNA level in AML, analyzed by LinkedOmics. **c** Survival curves of EFS in AML patients from the TCGA cohort

We investigated whether ARHGAP9 expression was associated with the prognosis of AML patients. The OS against ARHGAP9 expression was evaluated using the GEPIA and LinkedOmics databases. As shown on the GEPIA and LinkedOmics databases, high expression of ARHGAP9 was correlated with poor OS in AML (Fig. 3a, 3b). We further analyzed the survival data from the TCGA database and found ARHGAP9 overexpression was related to shorter event-free survival (EFS) (Fig. 3c), albeit statistical significance was not achieved.

**Fig. 4:**
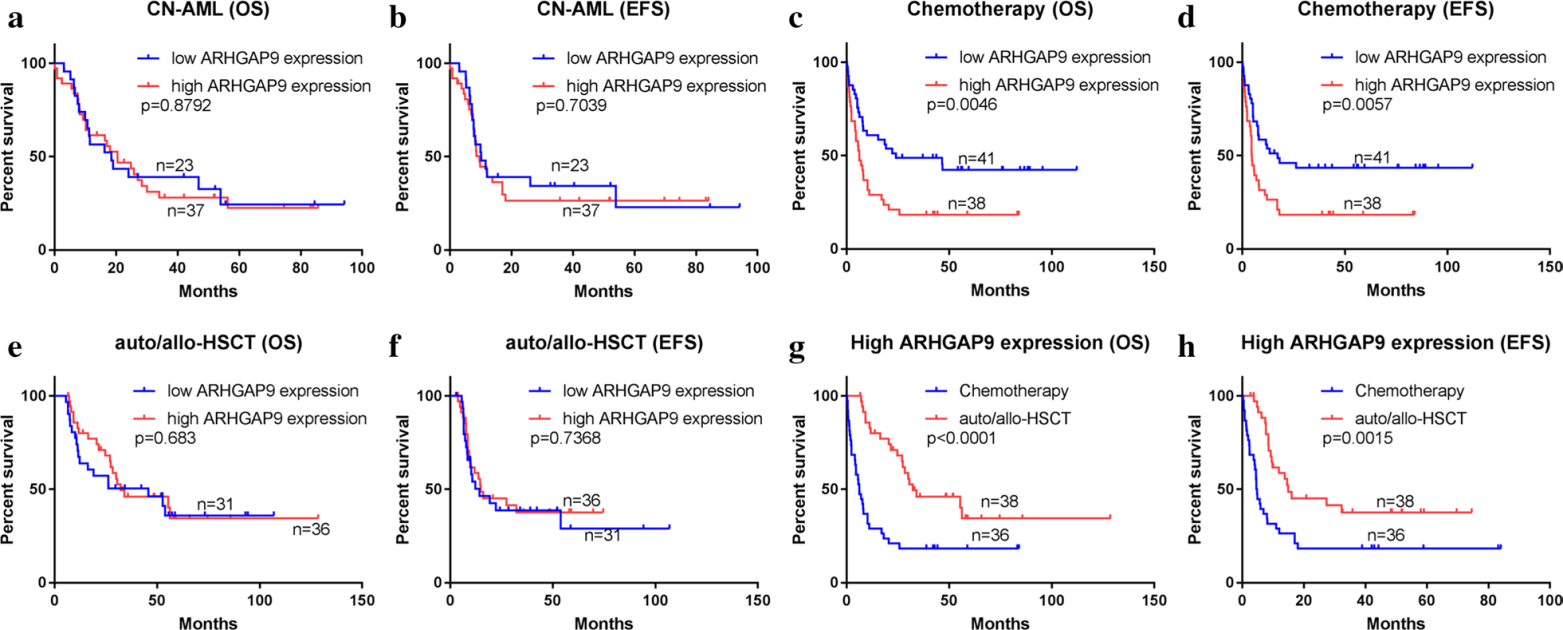
Prognostic values of ARHGAP9 with different factors on the survival of AML patients from the TCGA cohort. **a** OS and **b** EFS in CN-AML. **c** OS and **d** EFS of patients undergoing chemotherapy. **e** OS and **f** EFS of patients treated with auto/allo-HSCT. **g** OS and **h** EFS of patients in the ARHGAP9^high^ group

No significant difference in OS (Log-rank p = 0.8792) and EFS (p = 0.7039) was found between the ARHGAP9^low^ and ARHGAP9^high^ groups in CN-AML (Fig. 4a, b). To determine whether AML patients with high expression of ARHGAP9 could benefit from chemotherapy or auto/allo-HSCT, we divided the tested AML patients into two groups by their treatment regimens. Significantly shorter OS (Log-rank p = 0.0046) and EFS (Log-rank p = 0.0057) were found in AML patients who received with only chemotherapy (Fig. 4c, d). Among the patients who received auto/allo-HSCT, no significant differences in OS (Log-rank p = 0.6830) and EFS (Log-rank p = 0.7368) were found between the ARHGAP9^low^ and ARHGAP9^high^ groups (Fig. 4e, f). Patients undergoing auto/allo-HSCT had higher OS (p < 0.0001) and EFS (p = 0.0015) than those who received only chemotherapy (Fig. 4g, h). Acute promyelocytic leukemia (APL) (FAB M3) is a unique subtype of AML associated with peculiar clinical features and treatment strategies and the prognosis of APL is good. The prognostic values of ARHGAP9 expression in CN-AML, chemotherapy, and auto/allo-HSCT after the exclusion of FAB M3 from the AML cases were the same as those without the exclusion (Additional file 2: Figure S1), which indicated that the aforementioned prognosis results were not subject to the effect of FAB M3.

Overall, ARHGAP9 expression is a poor factor for AML rather than CN-AML. Moreover, chemotherapy alone showed no prognostic influence on ARHGAP9^high^ cases. And AML patients with high expression of ARHGAP9 could benefit from auto/allo-HSCT.

### ARHGAP9-associated gene analysis between ARHGAP9^high^ and ARHGAP9^low^ in AML patients

**Fig. 5:**
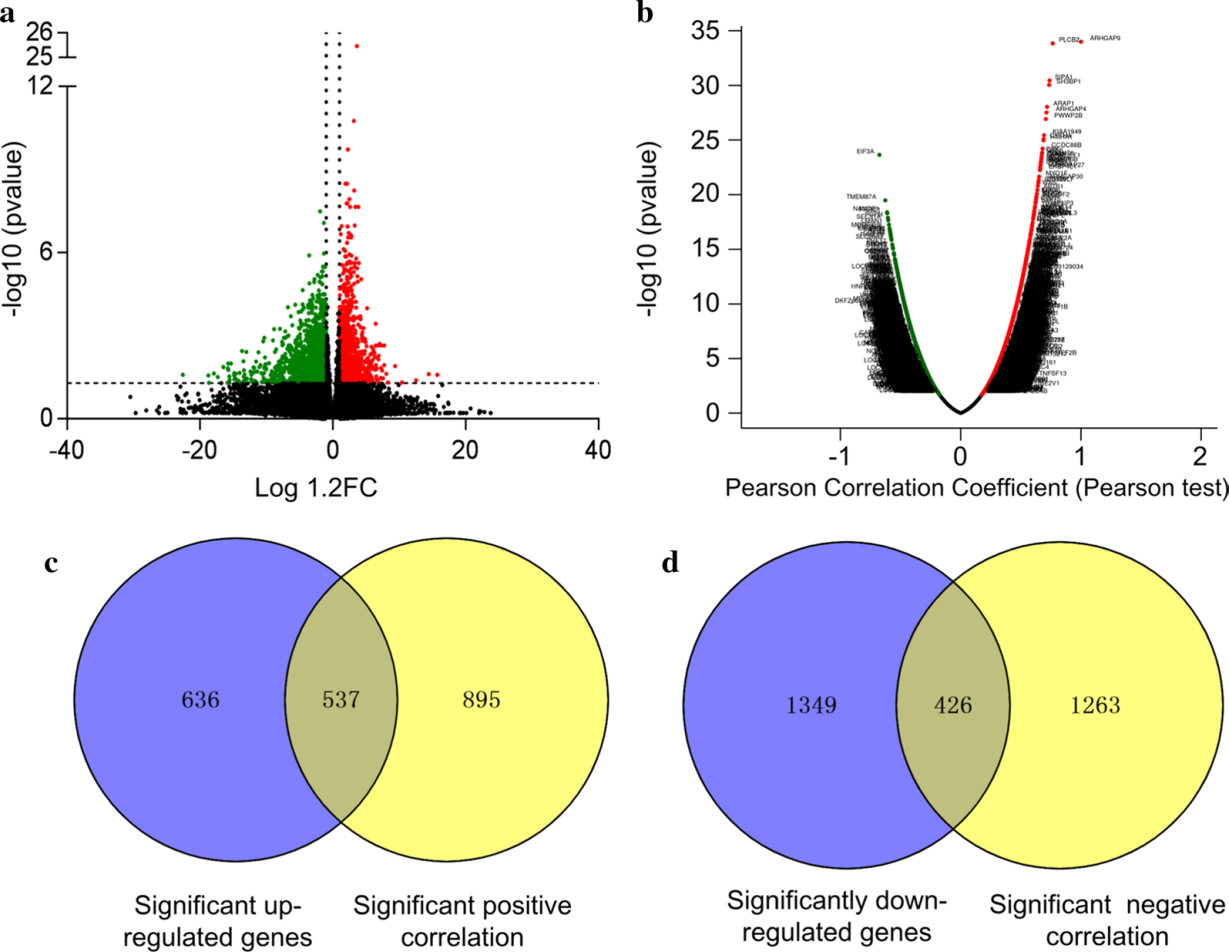
Genome-wide genes associated with ARHGAP9 expression. **a** Volcano plot of different gene-expression profiles between the ARHGAP9^low^ and ARHGAP9^high^ groups. **b** Volcano plots for the analysis of the co-expression genes associated with ARHGAP9 expression (LinkedOmics). **c** Overlapping genes between positively correlated genes and significantly increased genes. **d** Overlapping genes between negatively correlated genes and significantly reduced genes

To further explore the role of ARHGAP9 in AML, we firstly compared the transcriptomes of the ARHGAP9^low^ and ARHGAP9^high^ groups base on the TCGA database. A total of 2,948 genes were identified as significantly different between the ARHGAP9^low^ and ARHGAP9^high^ groups (p ≤ 0.05, |log1.2 FC|≥ 1, Additional file 1: Table S3); 1173 genes denoted by red circles and 1,775 genes denoted by green circles, were significantly upregulated and downregulated in the ARHGAP9 high group, respectively (Fig. 5a).

Subsequently, we analyzed the co-expressed genes in conjunction with the ARHGAP9 genes by using the LinkedOmics database. As shown in Fig. 5b, a total of 3,310 genes represented by dark red dots were positively correlated with ARHGAP9 expression, and 4,268 genes represented by dark green dots were negatively correlated with ARHGAP9 in AML. Among the genes correlated with ARHGAP9 expression, 3,121 co-expression genes were significant correlations with ARHGAP9 in AML (False discovery rate, FDR ≤ 0.05, p ≤ 0.05, and |cor.|≥ 0.3; Additional file 1: Table S4).

Comparison of the significantly different genes and co-expressed genes led to the determination of 963 overlapping genes. These 963 genes contained 537 positively upregulated genes and 426 negatively downregulated genes (Fig. 5c, d and Additional file 1: Table S5). The overlapping genes were used for subsequent studies.

### Functional analysis of the overlapping genes

**Fig. 6:**
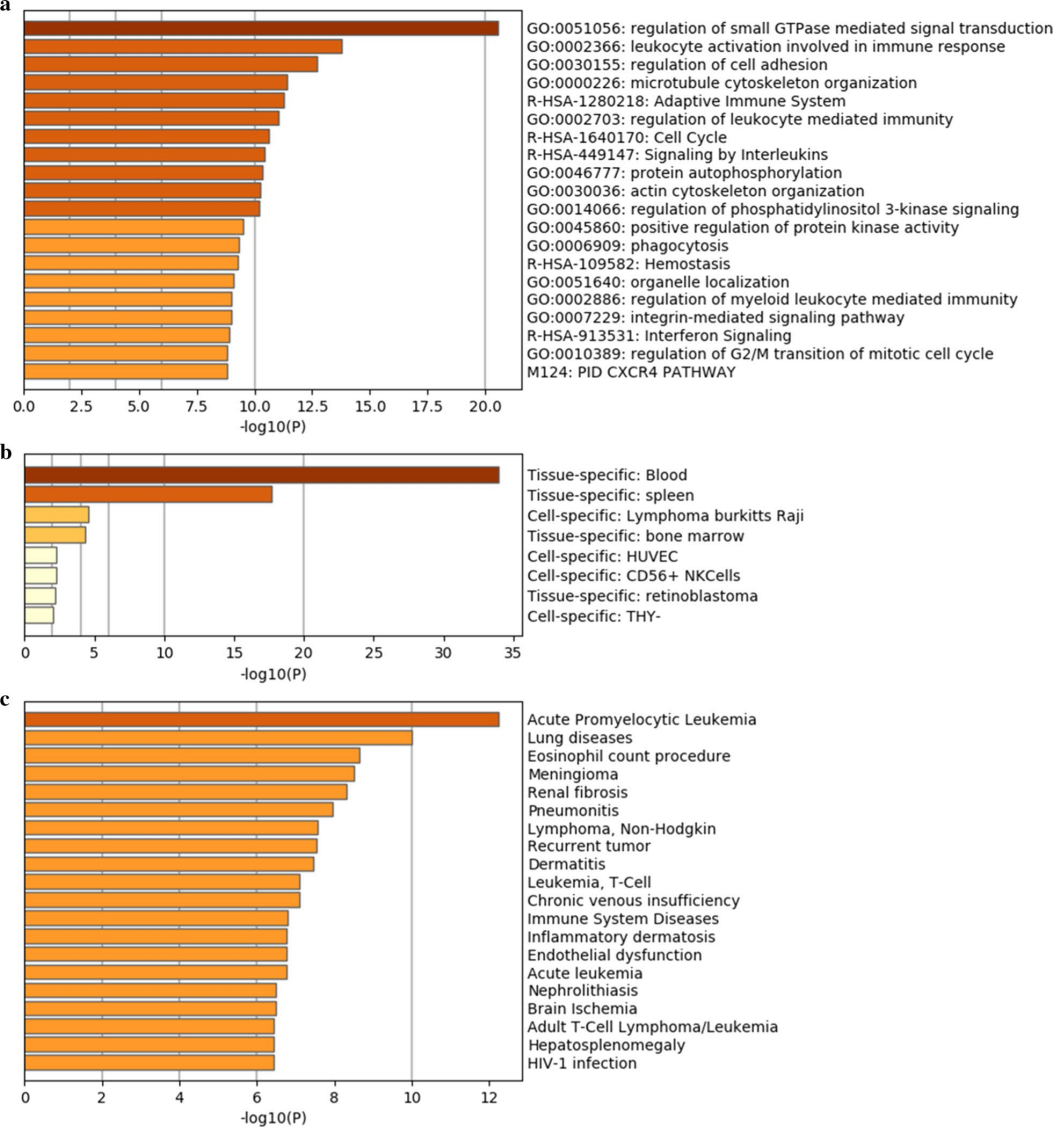
Enrichment of functions and signaling pathways of the overlapping genes in AML. **a** Analysis of GO and KEGG pathway associated with ARHGAP9 expression. **b** Enrichment of differentially expressed genes in tissues and cells. **c** Enrichment of overlapping genes in diseases

We then sought to investigate the possible biological function of ARHGAP9 in patients with AML. The 963 genes were analyzed by using tools in Metascape to study KEGG and GO annotation. The top 20 clusters of enriched sets are shown in Fig. 6a. ARHGAP9 mediated the function of Rho GTPases, including the regulation of small GTPases mediated signal transduction, regulation of cell adhesion, actin cytoskeleton organization, cell cycle, microtubule cytoskeleton organization, organelle localization, phagocytosis and regulation of G2/M transition of mitotic cell cycle. Notably, seven clusters belonged to the immune system including leukocyte activation involved in the immune response, signaling by interleukins, the adaptive immune system, regulation of leukocyte-mediated immunity, regulation of myeloid leukocyte-mediated immunity, interferon signaling, and interferon signaling. ARHGAP9 expression was also associated with protein autophosphorylation, regulation of phosphatidylinositol 3-kinase signaling (PI3K), positive regulation of protein kinase activity, CXCR4 pathway, and hemostasis. In addition, the overlapping genes were enriched in blood, spleen, and bone marrow (Fig. 6b), further suggesting immunological function of ARHGAP9 in leukemogenesis. Moreover, among different diseases, the overlapping genes participated primarily in APL (Fig. 6c).

### Validation of hub genes

**Fig. 7:**
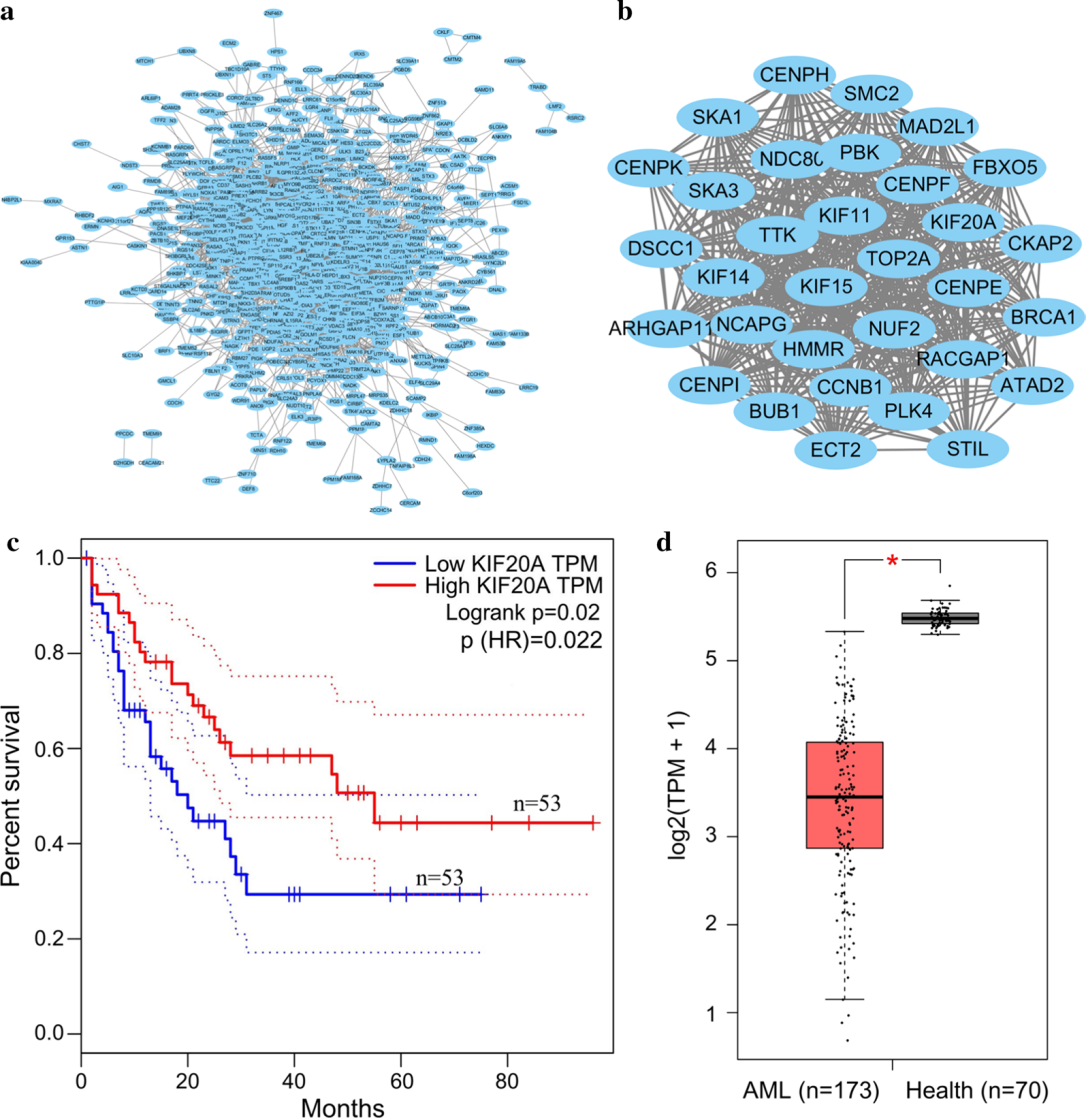
PPI network of the overlapping genes. **a** Network view of PPI for the overlapping genes. **b** MCODE analysis of the most significant interactions from **a** (MCODE score = 29.806); **c** Prognostic value of hub genes in AML, analyzed by GEPIA. **d** KIF20A expression in AML patients and healthy tissues, analyzed by GEPIA

The PPI network was constructed using the String website and the figures were generated by Cytoscape (MCODE plug-in). A total of 963 common genes were imported into the PPI network. We obtained 881 nodes and 5103 edges (Fig. 7a). The most significant module (MCODE score = 29.613) contained 32 genes. These genes are identified in Fig. 7b. Subsequently, OS analysis of the hub genes was performed using the GEPIA database. Of the 32 hub genes, 31 genes exerted no effect on OS in AML patients. Only KIF20A was significantly related to OS in AML (p = 0.02, Fig. 7c). KIF20A in the AML samples was significantly lower than that in normal tissues (Fig. 7d).

## Discussion

Rho GAPs inactivate Rho GTPases by the conservative GAP domain that promotes GTP hydrolysis and accelerates intrinsic GTPase activity, limiting the duration of the regulated reaction [29, 30]. Numerous reports suggest that the abnormal expression of Rho GAPs is observed in various tissues from patients with cancer and immunological diseases [31–34]. The expression of Rho GAPs is found to be heterogeneous among different tumors. For instance, ARHGAP30 was downregulated in lung cancer and colorectal cancer but overexpressed in pancreatic cancer [35–37]. Low expression of ARHGAP9 was found in hepatocellular carcinoma and bladder cancer, whereas high expression of ARHGAP9 was observed in breast cancer [17, 19, 38]. In the current study, ARHGAP9 is overexpressed in both AML samples and cell lines compared, relative to that in their normal counterparts [19]. Nonetheless, the low expression of ARHGAP9 was found in t(15; 17) AML.

Several studies investigating the role of Rho GAPs in cancer have found that most Rho GAPs were associated with good outcomes in various kinds of solid tumors [33, 36, 37, 39]. ARHGAP9 also showed a good prognosis in bladder cancer, hepatocellular carcinoma, and gastric cancer [17, 18, 38]. However, we found that, ARHGAP9 overexpression was associated with a poor prognosis in AML. These results suggest that ARHGAP9 can play different roles in various cancers. Meanwhile, DOCK2 belonging to the Rho GEFs family activates GTPase, which has a function contrary to that of Rho GAPs. DOCK2 was also an independent favorable prognostic factor for both EFS and OS in AML [40]. Thus, some regulators of Rho GTPase may have other functions in cancer, in addition to being associated with GTPase.

Despite the association of the high expression of ARHGAP9 with CN-AML, no relationship between ARHGAP9 expression and prognosis of CN-AML was found in the current study. In addition, several studies have shown that abnormal expression of some genes such as *NCALD, IL2RA*, and *BCL2* are associated with prognosis in AML patients with auto/allo-HSCT and/or chemotherapy [41–43]. Our findings showed that ARHGAP9^high^ groups had poor prognosis in post-chemotherapy AML patients, whereas no significant differences in OS and EFS were found between the ARHGAP9^high^ group and ARHGAP9^low^ group in patients who underwent after auto/allo-HSCT. These results suggest that the effects of ARHGAP9 over-expression can be eliminated by auto/allo-HSCT, instead of chemotherapy.

Rho GAPs, regulators of Rho GTPases, are expressed mainly in hematopoietic cells [8, 44–46]. Costa et al. showed that inadequate ARHGAP15 leads to enhanced chemotactic responses, straightened directional migration, amplified reactive oxygen species production, increased phagocytosis, as well as improved bacterial killing in neutrophils [44]. ARHGAP25 was found to negatively regulate leukocyte transendothelial migration in mice and phagocytosis in human neutrophilic granulocytes [45, 47]. Study in vitro and in vivo indicated that ARHGAP21 knockdown could impair the function of T cells, reduce erythroid commitment and differentiation, and enhance RhoC activity [12]. ARHGAP19, a hematopoietic-specific Rho GAP, affected the stiffness and shape of lymphocytes by regulating cytokinesis and chromosome segregation in T lymphocytes [8]. These combined findings suggest that Rho GAPs play an important role in hematopoietic cells and regulate cell motility, cell cycle, adhesion, phagocytosis, NADPH oxidase, auto/allo-HSCT development, inflammatory responses, and neutrophil chemotaxis. Consistent with the previously published reports, the present study showed that differentially expressed genes associated with ARHGAP9 expression were involved in microtubule cytoskeleton organization, actin cytoskeleton organization, phagocytosis, regulation of cell adhesion, cell cycle.

Although the other members of the Rho family GAPs were shown to be involved in several vital functions of neutrophils, our study showed that differentially expressed genes associated with ARHGAP19 expression were enriched in APL. Meanwhile, 95% of APL consisted of the abnormalities of t(15;17), which encodes the promyelocytic leukemia–retinoic acid receptor alpha (PML-RARA) fusion protein [48]. Moreover, ARHGAP9 expression was the lowest in t(15;17) AML among other chromosome abnormalities in AML, and all patients with t(15;17) were in the ARHGAP9^low^ group in the current study. Therefore, ARHGAP9 expression may be suppressed by the PML-RARA fusion protein. The physiological role of ARHGAP9 in APL requires further investigation..

Upon immunization, Rho GAPs are critical for innate immunity and adaptive immunity [49–51]. Meanwhile, more than 11 members of Rho GAPs participate in various neutrophil functions that belong to adaptive immunity [52]. Given that the overlapping genes were mainly enriched in the immune system and immune tissues, we speculated that ARHGAP9 plays a significant role in the immune response in AML. The phosphorylation of Rho GTPase not only affects Rho GTPase activity by altering their conformation, but also regulate Rho GTPase ability to interact with the effector proteins, and thereby influencing their subcellular localization [53–55]. This finding would explain the enrichment of some overlapping genes in protein autophosphorylation, positive regulation of protein kinase activity, regulation of PI3K, and organelle localization, which displayed the activity of ARHGAP9 may be regulated by the phosphorylation in the AML.

## Conclusions

High expression of ARHGAP9 was found in AML tissues and cells, and elevated ARHGAP9 was significantly correlated with poor outcome in AML. Auto/allo-HSCT, rather than chemotherapy, can overcome the adverse outcomes related to high ARHGAP9 expression. Functional analysis of differentlially expressed genes between those exhibiting high and low expression of ARHGAP9 indicated that ARHGAP9 could perform multiple functions in AML. The physiological role of ARHGAP9 in AML requires further study. Moreover, ARHGAP9 was downregulated in the t(15;17) patients, and differentially expressed genes associated with ARHGAP9 expression were enriched in APL. PML-RARA may act as negative regulators of ARHGAP9 expression in AML.

## Supplementary Information

**Additional file 1.** Additional tables.

**Additional file 2:** Figure S1. The prognostic values of ARHGAP9 with different factors on the survival of AML patients without FAB M3 from the TCGA cohort. a OS and b EFS of CN-AML without FAB M3. c OS and d EFS in AML patients undergoing chemotherapy without FAB M3. e OS and f EFS in patients treating with auto/allo-HSCT without FAB M3. g OS and h EFS of patients without FAB M3 in ARHGAP9high group.OCX 178 KB).

## Data Availability

The datasets used and/or analyzed during the current study are available. Please contact the author to get the datasets.

## Abbreviations

ARHGAP9: Rho GTPase activating protein 9
AML: Acute myeloid leukemia
APL: Acute promyelocytic leukemia
CN-AML: Cytogenetic normal acute myeloid leukemia
TCGA: The Cancer Genome Atlas
EFS: Event-free survival
OS: Overall survival
FAB: French-American-British subtypes
BM-blast: Bone marrow blast
PB-blast: Peripheral blood blast
WBC: White blood cell
allo-HSCT: Allogeneic hematopoietic stem cell transplantation
RNA-Seq: RNA sequencing
GEO: Gene Expression Omnibus
CCLE: Cancer cell cine encyclopedia
HPA: Human Protein Atlas
PPI: Protein–protein interaction
GEFs: Guanine nucleotide exchange factors
GAPs: GTPase activating proteins
GO: Gene ontology
KEGG: Kyoto encyclopedia of genes and genomes
n: Number of patients
FAB: French–American–British subtypes
BM-blast: Bone marrow blast
PB-blast: Peripheral blood blast
WBC: White blood cell
WBC: Acute promyelocytic leukemia
PML-RARA: Promyelocytic leukemia–retinoic acid receptor alpha
PI3K: Phosphatidylinositol 3-kinase signaling
