# Supplementary material for: The role of ARHGAP9: clinical implication and potential function in acute myeloid leukemia
Source: J Transl Med. 2021 Feb 12;19:65. doi: 10.1186/s12967-021-02733-5 (PMC7881617; doi:10.1186/s12967-021-02733-5)
Supplement: Supplementary file 2 — Additional file 2: Figure S1. The prognostic values of ARHGAP9 with different factors on the survival of AML patients without FAB M3 from the TCGA cohort. a OS and b EFS of CN-AML without FAB M3. c OS and d EFS in AML patients undergoing chemotherapy without FAB M3. e OS and f EFS in patients treating with auto/allo-HSCT without FAB M3. g OS and h EFS of patients without FAB M3 in ARHGAP9high group.OCX 178 KB). [file 12967_2021_2733_MOESM2_ESM.docx]

**Additional file 2: Figure S1** The prognostic values of ARHGAP9 with different factors on the survival of AML patients without FAB M3 from the TCGA cohort. **a** OS and **b** EFS of CN-AML without FAB M3. **c** OS and **d** EFS of AML patients undergoing chemotherapy without FAB M3. **e** OS and **f** EFS of patients treating with auto/allo-HSCT without FAB M3. **g** OS and **h** EFS of patients without FAB M3 in ARHGAP9^high^ group

**
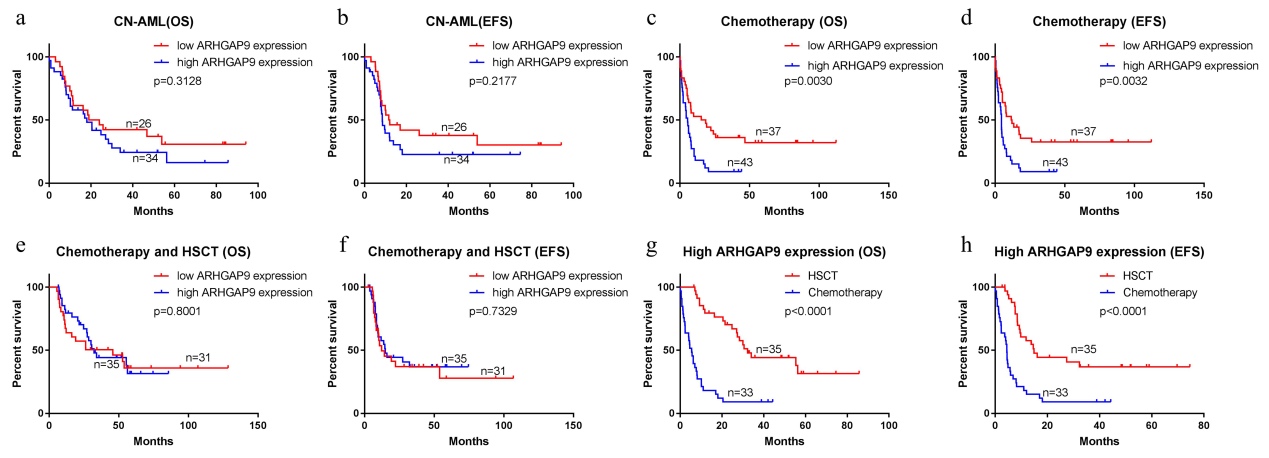
**
